# Neural Network Modelling of Track Profile in Cold Spray Additive Manufacturing

**DOI:** 10.3390/ma12172827

**Published:** 2019-09-02

**Authors:** Daiki Ikeuchi, Alejandro Vargas-Uscategui, Xiaofeng Wu, Peter C. King

**Affiliations:** 1 School of Aerospace, Mechanical and Mechatronic Engineering, The University of Sydney, Sydney, NSW 2006, Australia; 2 Commonwealth Scientific and Industrial Research Organisation Manufacturing, Private Bag 10, Clayton, VIC 3169, Australia

**Keywords:** cold spray, neural network, additive manufacturing, model, spray angle, profile

## Abstract

Cold spray additive manufacturing is an emerging technology that offers the ability to deposit oxygen-sensitive materials and to manufacture large components in the solid state. For further development of the technology, the geometric control of cold sprayed components is fundamental but not yet fully matured. This study presents a neural network predictive modelling of a single-track profile in cold spray additive manufacturing to address the problem. In contrast to previous studies focusing only on key geometric feature predictions, the neural network model was employed to demonstrate its capability of predicting complete track profiles at both normal and off-normal spray angles, resulting in a mean absolute error of 8.3%. We also compared the track profile modelling results against the previously proposed Gaussian model and showed that the neural network model provided comparable predictive accuracy, even outperforming in the predictions at cold spray profile edges. The results indicate that a neural network modelling approach is well suited to cold spray profile prediction and may be used to improve geometric control during additive manufacturing with an appropriate process planning algorithm.

## 1. Introduction

Cold spray is a materials deposition technology that is suitable for coatings and repair and is widely employed in industrial applications. This technology adopts a supersonic gas jet to accelerate powder particles to 500–1000 m/s and enables solid-state deposition onto a substrate by kinetic energy of the particles without melting. This mechanism offers unique characteristics that are difficult to achieve otherwise, including: Low oxygen-content deposition, the avoidance of melting-induced microstructure changes, and the ability to handle oxygen-sensitive materials without a protective atmosphere [1,2,3]. Furthermore, a high deposition rate can be achieved with a narrow nozzle diameter, resulting in a well-defined and high-density particle beam at small standoff distances [4].

The characteristics of cold spray are now recognized to offer great potential as an alternative solution to the field of additive manufacturing, namely Cold Spray Additive Manufacturing (CSAM) [5,6,7,8,9]. The elimination of a protective atmosphere environment provides the ability to fabricate larger manufactured components that are not possible with other additive manufacturing technologies, e.g., powder-bed additive manufacturing, while still allowing for excellent flexibility in the selection of oxygen-sensitive powder materials [9,10,11]. This benefit of cold spray technology can be further enhanced by the inclusion of a robotic system in CSAM which also allows the stability of fabrication, more Degrees of Freedom (DoF) for complex shapes and industrial automation [12,13,14]. Such robotic CSAM effectively utilizes its high deposition rate to produce components at industrially relevant part turnaround times [7,9]. Owing to the benefits of CSAM, successful demonstrations have been reported largely in aerospace industries at different levels of fabrication complexity: Simple rotational structures [15,16] and more complex components (e.g., fin arrays) [17,18,19].

However, the CSAM technology has not yet reached a mature technology level where it is considered a viable and reliable replacement to what is currently in use in commercial manufacturing industries due to a number of fundamental and practical problems. Fundamental problems are associated with the acceptable range of CSAM materials selection [20,21] and the microstructure and mechanical properties of deposits under different process parameters [22,23]. In contrast, practical challenges attract less attention from the CSAM community although providing a solution to them is a key aspect to facilitating the development of a commercial CSAM technology. One such practical challenge is the geometric control of as-fabricated components often associated with the nature of high production rate additive manufacturing technologies: namely, CSAM [8,9,24], Wire and Arc Additive Manufacturing (WAAM) [13,25] and Laser Cladding (LC) [26,27]. Low geometric control is attributed to a range of key issues that limit the application of additive manufacturing technologies such as the necessity of post-machining, difficulty in fabricating complex shapes, geometry-induced property variations and inconsistent quality of fabricated parts [8,9,28]. Therefore, addressing the challenge of geometric control is undoubtedly of great importance in CSAM as well as other high-speed additive manufacturing technologies.

From the perspective of geometric control, the development of a high-accuracy process model on the smallest processing unit (e.g., single cold spray track) offers a promising solution to the aforementioned problem since an aggregate of single tracks determines final part geometry. Furthermore, such a single-track model often plays a key role in the modelling of higher processing unit (i.e., overlapping and overlayer models) in the literature [25,29]. Previous studies of the single-track modelling fell into two main approaches: mathematical and data-driven modelling.

Suryakumar et al. approximated the profile of a single symmetric bead as a parabolic model in WAAM [30]. The model was developed in terms of WAAM process parameters as well as bead geometric characteristics (i.e., height and width). A second-order regression model was established with the aid of experiments to express the bead height in terms of the process parameters from which the bead width was calculated mathematically. This hybrid modelling approach showed reasonable pictorial agreement with a verification bead profile under the reported experimental conditions. Cai et al. employed a Gaussian model with a constant scaling coefficient to approximate the profile of a single symmetric cold spray track under different standoff distance scenarios [31]. The authors integrated the derived model into their Thermal Spray Toolkit, a software package in ABB RobotStudio^®^, for offline programming to predict cold spray track profiles.

Alternatively, a data-driven modelling approach attracted attention as an alternative to mathematical modelling approach with the increased accessibility of available software options. Mahapatra and Li applied an Artificial Neural Network (ANN) modelling with back propagation algorithm to predict the cross-sectional geometry of a single symmetric track profile in highly nonlinear and multivariate nature Pulsed-Laser Powder Deposition process [32]. The trained ANN model predicted bead width, cross-sectional area and heights at three segments within mostly 10% mean absolute error. Xiong et al. highlighted the development of ANN and second-order regression models in single symmetric bead geometry prediction in WAAM [33]. The authors compared the performance of the developed models in bead height and width predictions and reported that the ANN model outperformed in both predictions due to its ability to approximate any nonlinear process.

Despite the great capability of ANN modelling as seen in other additive manufacturing processes, it has drawn only a small amount of interest as a track modelling approach from the CSAM community. Furthermore, the application of the ANN modelling in prediction was greatly limited to key geometric characteristics only, e.g., height and width, in additive manufacturing [27,33]; such observations formed an underlying motivation to study in mathematical modelling that could describe more detailed geometric track profiles. This trend can be seen in previous CSAM studies focusing on the mathematical approach only (i.e., Gaussian model) to predict a single-track profile at both normal and off-normal spray angles [24,34]. However, a data-modelling approach can be more competent than what was previously conceived of in additive manufacturing as recently demonstrated successfully by Kochar et al. in joining application [35]. The data-driven approach offered great nonlinear mapping capability and multi-output predictions with affordable model complexity; such advantages are particularly desirable as asymmetric track profiles resulting from off-normal spray angles have become more frequent due to the necessity of complex spray strategies in CSAM. An accurate modelling of both symmetric and asymmetric single-track profiles with high geometric details will contribute to the improved geometric control in CSAM, enabling the fabrication of more complex and consistent parts with minimal post-machining.

In this study, we focus on the modelling of a single-track profile with high morphology in CSAM, both at normal and off-normal spray angles, using an ANN modelling to demonstrate its potential as a predictive modelling approach in additive manufacturing. The significance of this study is three-fold: (1) The application of a data-driven modelling approach in the prediction of a track profile to CSAM, (2) the modelling of an asymmetric track profile using the ANN model instead of the previous mathematical approach, and (3) the ANN modelling of a detailed track profile rather than key geometric characteristics only.

## 2. Materials and Methods

An ANN is a type of data-driven model for supervised machine learning which is sufficiently capable of handling nonlinearity and constructing an input–output relationship mapping based on a set of training data. The development of an effective ANN relies on a number of key design aspects such as input variable selection, data quality and network architecture [36,37]. In this study, three process variables were chosen as the inputs of the ANN model: spray angle, traverse speed, and standoff distance. These process variables are precisely controllable in real time with the support of an appropriate robotic system [12] and have been shown to be influential on cold spray geometric profiles in previous studies [24,31].

In this study, a full factorial approach was adopted to define the values of the input variables in the ANN training dataset due to the nonlinear and complex nature of CSAM and the affordable number of the input variables. In this approach, three levels were defined for traverse speed and standoff distance, while four levels were employed to capture the effects of spray angle on track profiles more precisely. The values of the input variables at each level are listed in Table 1. The lowest- and highest-level values were determined as those of the corresponding operating limits to maintain the sufficient deposit quality in the CSAM system. Defining the parameter boundaries at these operating limits avoided the weakness of an ANN model in extrapolation outside the training dataset [38]. The input values of intermediate levels were equally spaced between the lowest and highest level such that possible interactions between the input variables were adequately captured [39]. The resulting experiment design matrix required the fabrication of 36 samples for the ANN training dataset. The details of the experiment design matrix of each sample can be found in the Supplementary Materials (i.e., Tables S1 and S2).

### 2.1. Sample Preparation

All sample preparations were performed using a commercial Impact Innovations (Haun, Germany) 5/11 cold spray gun guided by an ABB (Zurich, Switzerland) 4600 6-DoF robot. The cold spray gun was equipped with a long pre-chamber and an Impact Innovation’s OUT1 tungsten carbide de Laval nozzle with a 6.2 mm exit diameter. Commercial purity grade-2 titanium from AP&C (Boisbriand, Canada) was selected as the powder feedstock. The particles were prepared by gas atomization and distributed within the size of 15 to 45 µm: (i.e., D_10_ = 19 µm, D_50_ = 34 µm and D_90_ = 45 µm). Nitrogen gas was preheated to 600 °C at a pressure of 5 MPa to accelerate the particles that were fed into the upstream of the nozzle at a feed rate of 1.9 kg/h. These spray variables except those listed in Table 1 were held constant throughout the sample fabrications. The substrate was a strip of commercial purity grade-2 titanium with a dimension of 6 × 30 × 200 mm. The surface of the substrate was prepared with a milling machine from Avemax Machinery (Taichung City, Taiwan) followed by grinding with P120-SiC emery paper from LECO (Moenchengladbach, Germany). Ethanol was used to clean the surface prior to the sample fabrications. The fabrication of all samples was randomized to obtain statistically unbiased results and minimize the effects of potential extraneous factors [40]. RobotStudio^®^ software version 6.08 (ABB Robotics, Zurich, Switzerland) was used to verify that there was sufficient travel past the edge of the substrate to allow for the robot trajectory and traverse speed to stabilize prior to sample fabrications.

The profile of each sample was measured five times at randomly selected locations using a LEXT OLS4100 confocal laser scanning microscope from Olympus (Tokyo, Japan) and scanControl 2950-100 laser scanner from Micro-Epsilon (Ortenburg, Germany) with the z-axis measuring precision of at least 12 µm. The obtained measurements were processed with the in-built filtering: Flat Surface filtering in LEXT OLS4000 and average filtering with a filter size of 7 in scanControl Configuration Tools 6.0. The filtered profiles were averaged for each sample, resulting in the ANN output profiles considered in this study.

### 2.2. Artificial Neural Network Model Design and Training

In this study, a static multilayer perceptron ANN model was considered due to various successful demonstrations of its application as a predictive model in manufacturing processes. The model consisted of three different layer types: input layer, hidden layer, and output layer. Each layer contained a number of neurons with connections in between through activation functions. The number of neurons in the input layer corresponded to the number of input variables considered, i.e., 3 neurons in this study. The neurons in the hidden layer act as computational elements processing nonlinear mapping between the input and output variables and largely influence the performance and reliability of an ANN model [41]. Although a higher number of hidden neurons allow more accurate predictions or the modelling of more complex processes, it poses a higher risk of overfitting that is critical with only 36 training samples. For developing a reliable ANN model, this study iteratively investigated the performance of the ANN model with the different number of hidden neurons (i.e., 1 to 15 neurons) for each hidden layer. Similarly, the number of hidden layers was incrementally changed between 1 to 3 layers to optimize the ANN model architecture. Furthermore, the number of output neurons must be sufficient to achieve the objective of modelling a detailed track profile in CSAM. We adopted the area validation methodology proposed by Kochar et al. [35] in which polar lengths were considered as output neurons, measured from the tool center point as the origin. The number of output neurons was incrementally changed from 5 neurons with equal angular spacing between each output neuron (e.g., 45° each between 5 neurons) until the sufficient number was reached. Here, the sufficient number of output neurons was defined as that whose enclosed area reached and maintained at least 99% of the sample cross-sectional area in the last five consecutive candidates. An activation function is another critical aspect in an ANN model that computes the output of a neuron given the set of weights and biases as inputs. In this study, the commonly used hyperbolic tangent sigmoid and linear activation functions were chosen for hidden and output layers respectively. With the selected activation functions, all inputs and outputs variables were scaled to [−1 1] for improved training process [41].

The back-propagation algorithm with Bayesian regularization was selected as the training function of the ANN model. This algorithm depends on Levenberg-Marquardt optimization for updating weights and biases. The benefits of this training algorithm are two-fold: Robustness and the elimination of validation dataset, reducing the number of samples required [42]. In addition, the performance of the training process was measured using Mean Squared Error (MSE). The training of the ANN model was conducted using Deep Learning Toolbox in MATLAB^®^ version R2018a and the training dataset in the supplementary material (i.e., Samples 1 to 36 in Table S1). To avoid the effects of different initial weights and biases, each ANN candidate model was retrained 100 times. 

The performance of the trained ANN model was evaluated using an independent set of testing samples (see Table S2 in the Supplementary Material). The number of testing samples was determined according to the 75-25 training-testing data division method [43], resulting in a total of 12 testing samples (i.e., Samples 37-48). The values of the input variables in the testing dataset were randomly selected between their minimum and maximum operating limits with the aid of MATLAB^®^ version R2018a.

## 3. Results and Discussion

### 3.1. Single-Track Profiles Validation

**Figure 1:**
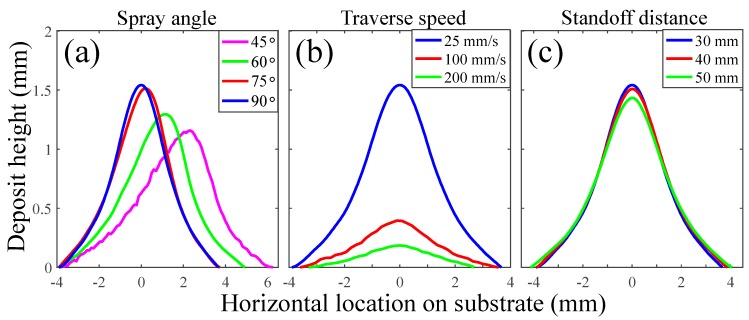
(**a**) the effect of spray angle at 25 mm traverse speed and 30 mm standoff distance (45°—sample 1, 60°—sample 2, 75°—sample 3, 90°—sample 4), (**b**) the effect of traverse speed at 90° spray angle and 30 mm standoff distance (25 mm/s—sample 4, 100 mm/s—sample 8, 200 mm/s—sample 12) and (**c**) the effect of standoff distance at 90° spray angle and 25 mm/s traverse speed (30 mm—sample 4, 40 mm—sample 16, 50 mm—sample 28).

The quality of the cold spray profile samples was validated against the previous CSAM studies in terms of the effects of the input variables on the sample profiles. Figure 1 shows the effects of the following input variables on the profiles of the selected training samples: (a) Spray angle at 25 mm/s traverse speed and 30 mm standoff distance, (b) Traverse speed at 90° spray angle and 30 mm standoff distance, and (c) Standoff distance at 90° spray angle and 25 mm/s traverse speed. 

In Figure 1a, it is clear that the spray angle was positively correlated to the height and negatively to the width of the sample profiles, being consistent with the previous studies [24,44]. The smaller effect of spray angle between 75° and 90° was attributed to the smaller relative deposition efficiency drop; in comparison, such phenomenon was observed between 80° and 90° spray angle in [24].

Importantly, the effect of traverse speed was found to be nonlinear and the most influential on the track profiles in Figure 1b. This observation suggests that the more levels of traverse speed may be employed in the experimental design matrix for the ANN training dataset to integrate more relevant information into an ANN model, especially at low traverse speeds (i.e., between 25 and 100 mm/s). The lower traverse speed resulted in a thicker and sharper track profile, while widening the track profile as also seen in [9,24].

In contrast, the effect of standoff distance was the least influential on the track profiles in Figure 1c. With the larger standoff distance, the track profile became shorter and wider as observed in [31]. This phenomenon was previously confirmed by Pattison et al. [45] and indicated that the standoff distance parameter space covered in this study was in the medium region near the optimal deposition efficiency point. It is of great interest to study further towards the nonlinear extreme ends (e.g., 10 and 100 mm), maximizing the benefits of nonlinear mapping ability in an ANN model.

In summary, the validation of the selected track profiles confirmed that the fabricated profiles were consistent with the previously reported trends and therefore of sufficient quality as the output profile data considered in this study. Note that the track profiles of all samples are presented in Figures S1–S3 in the Supplementary Materials.

### 3.2. Neural Network Architecture Validation

**Figure 2:**
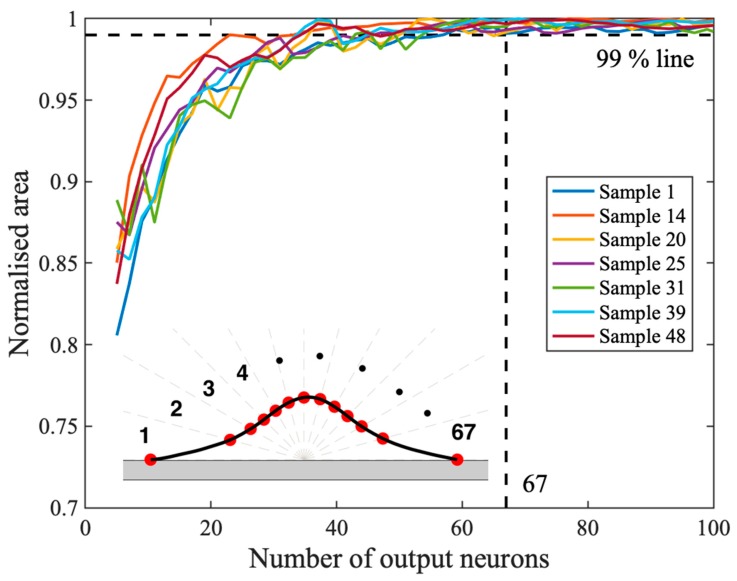
The validation results of the randomly selected samples. The validation of the number of output neurons indicates that the mean 67 output neurons are sufficient to describe the track profile with high fidelity.

The area validation method for determining the sufficient number of output neurons was performed over all the samples and the results of some randomly selected samples are shown in Figure 2 to illustrate the trend of area convergence. The mean sufficient number of output neurons was found as 67 over all the samples, while the maximum number was 167. Here, 67 output neurons were chosen for the ANN model, taking polar lengths from the tool center point at every 2.72°. This selection was because the maximum sufficient number of output neurons resulted in capturing too fine geometric features that could be considered as noises. Furthermore, the fewer number of output neurons allows a simpler ANN architecture, thereby reducing the computational burden of training process, while the resulting ANN model is accurate enough to achieve the objective of describing a detailed CSAM track profile. The resulting output neuron parameters are presented in Tables S3 and S4 in the Supplementary Materials.

**Figure 3:**
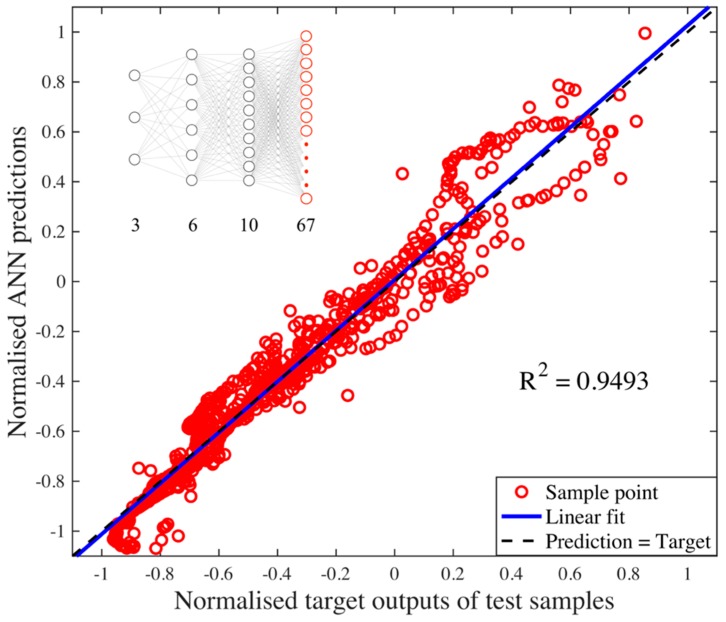
Normalized ANN predictions vs. target output neuron values of all test samples. The ANN had the architecture of [3 6 10 67], resulting in MSE of 0.009454 and R^2^ = 0.9493.

The iterative investigation of different ANN hidden layer topologies concluded that two hidden layers with 6 and 10 neurons achieved the best predictive performance on the normalized independent testing dataset with MSE of 0.009454 and R^2^ coefficient of 0.9493 (see Figure 3). The mean predictive performance for each output geometric point was evaluated among all 12 testing samples and the corresponding overall predictive performance for all 67 outputs is summarized in Table 2. Both Mean Absolute Percent Error (MAPE) and Maximum Absolute Percent Error (MXAPE) were reasonable in comparison with the previous studies in different manufacturing processes (i.e., MAPE = just below 10%, MXAPE ≈ 11% [35] and MAPE = 6.611%, MXAPE = 10.31% [46]). Consequently, the results demonstrated the suitability of a data-driven ANN modelling approach to the prediction of a track profile in CSAM.

### 3.3. Evaluation of Artificial Neural Network Modelling for Predicting Single-Track Profiles

**Figure 4:**
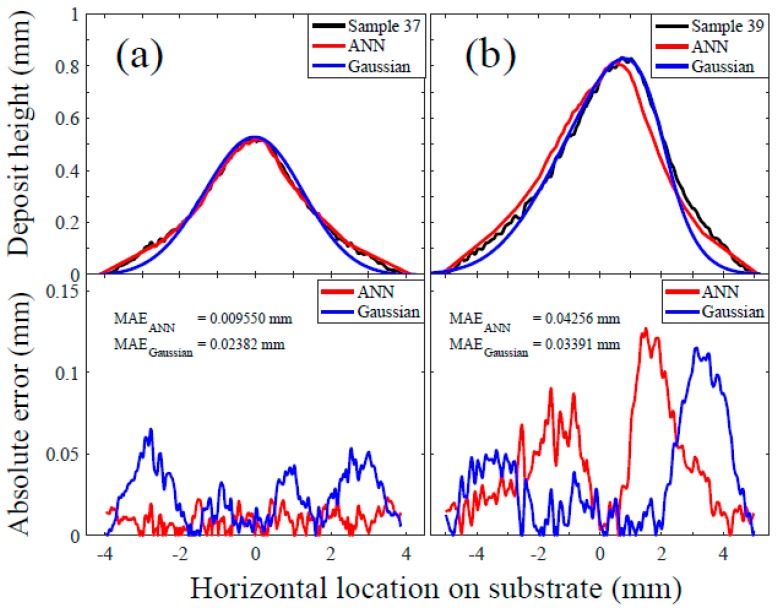
The experimental single-track profiles of the two selected samples are shown as illustrative examples along with the corresponding ANN (red) and Gaussian (blue) models: (**a**) Sample 37 (spray angle: 86°, traverse speed: 75 mm/s, standoff distance: 45 mm) and (**b**) Sample 39 (spray angle: 48°, traverse speed: 34 mm/s, standoff distance: 41 mm).

Figure 4 shows the track profile of the two selected test samples as an illustration: (a) Sample 37 at a nearly normal spray angle (i.e., 86°) and (b) Sample 39 at a spray angle of 48°. The developed ANN model was used to predict the track profiles, resulting in a qualitatively good agreement with the measured profiles. The MAEs were 0.009550 mm and 0.04256 mm for Sample 37 and 39, respectively. Thus, it is demonstrated that the application of an ANN modelling approach is possible to predict both symmetric and asymmetric track profiles at normal and off-normal spray angles. However, for Sample 39 at a lower spray angle, a larger deviation from the measured profile was found, as compared to Sample 37, in the high region of the track profile (between 3 and 7 mm on the substrate). The possible causes for this observation include: (1) The lack of training samples within this region to provide sufficient robustness to external factors (e.g., robot joint misalignment and tool centre point variation) at an off-normal spray angle and (2) inefficient experimental design matrix to capture high nonlinearities in CSAM, e.g., more traverse speed levels may be suitable as discussed in Section 3.1 towards the low-speed end, resulting in thicker track profiles. The robustness issue was also raised in the application of ANN modelling in welding [35], but it is more severe when a large number of ANN output predictions is necessary with a small number of input parameters such as in this study. Note that the ANN prediction results for all other test samples are presented in Table S5 and graphically shown in Figure S4 in the Supplementary Materials.

To demonstrate the potential of ANN modelling to predict detailed track profile for the objective of this study, in Figure 4, we also compared the ANN modelling results against the Gaussian modelling approach in cold spray proposed by Chen et al. [24]. The details of the Gaussian models can be found in Table S6 in the Supplementary Material. The ANN modelling approach showed about 2.5 times smaller MAE than the Gaussian model for Sample 37, but about 1.3 times larger MAE for Sample 39. The latter result was mainly attributed to the larger deviation at the high portion of the track profile as discussed previously. Meanwhile, the ANN modelling showed better predictive performance in the region of track profile edges than the Gaussian model. Such better predictive performance at profile edges was most likely due to the ANN model adequately capturing the complex multivariate nature of cold spray process (e.g., bow shock and compressed gas layer [47]), while this was observed to be lacking with the Gaussian model used previously in cold spray [24,31]. In summary, the comparative study of the two modelling approaches in Figure 4 showed that the ANN modelling possessed the potential to provide the prediction of detailed track profiles in CSAM at the same level of accuracy or higher.

## 4. Conclusions

This study demonstrated the potential of a data-driven modelling approach in the prediction of single-track profiles in CSAM, rather than only key geometric features as in previous studies. The ANN modelling enabled an accurate description of track profiles at even off-normal spray angles that are frequently encountered during the cold spray process of complex shapes. Furthermore, the detailed track profiles predicted by the ANN model were in good qualitative agreement with the measured profiles, even outperforming at the region of profile edges as compared to the previously proposed Gaussian modelling approach. Therefore, the data-driven modelling, in combination with an appropriate process planning algorithm, possesses the potential to improve the problem of geometric control in additive manufacturing processes and therefore foster the development of a commercial CSAM technology. With the appropriate adjustment of ANN input feature parameters and architecture, the approach presented in this study can be extended to other additive manufacturing techniques such as WAAM and LC.

However, the limitation of the ANN modelling approach was also encountered due to the size of training dataset and robustness. These issues were more significant in this study as the ANN approach adopted a larger number of output neurons than previous studies where only key geometric features were predicted. Therefore, it is of great importance in future works that a more data-efficient modelling approach is explored, and real-time measurement and a data processing system are developed so that the data diversity and collection rate increase. Furthermore, the comparative study of the two models showed that the Gaussian model predicted with better accuracy within the high portion of track profiles, while the ANN model was more accurate towards the profile edges. This finding triggers a motivation for exploring a hybrid modelling approach in future works, taking advantages from the two modelling approaches, while minimizing the disadvantages discussed in this study.

In addition to the aforementioned future works, we plan to extend this study to overlapping and overlayer modellings and integrate the ANN model from this study into our toolpath planning algorithm at a system level.

## Figures and Tables

**Table 1:** The levels of input variables in the experimental design matrix for the Artificial Neural Network (ANN) training dataset: 4 levels for spray angles, 3 levels for traverse speed and 3 levels for standoff distance.

Level	Spray Angle (°)	Traverse Speed (mm/s)	Standoff Distance (mm)
1	45	25	30
2	60	100	40
3	75	200	50
4	90	-	-

**Table 2:** Summary of the performance evaluation results of the developed model in Figure 3 in terms of Mean Absolute Error (MAE), Maximum Absolute Error (MXAE), Mean Absolute Percent Error (MAPE), and Maximum Absolute Percent Error (MXAPE).

MAE (mm)	MXAE (mm)	MAPE (%)	MXAPE (%)
0.05782	0.1522	8.342	10.20

## Supplementary Materials

The following are available online at https://www.mdpi.com/1996-1944/12/17/2827/s1, Figure S1: the measured track profiles of Sample 1 to 18, Figure S2: the measured track profiles of Sample 19 to 36, Figure S3: the measured track profiles of Sample 37 to 48, Figure S4: the predicted track profiles using the developed ANN model, Table S1: Input parameters in the training dataset, Table S2: Input parameters in the testing dataset, Table S3: Output parameters in the training dataset, Table S4: Output parameters in the testing dataset, Table S5: ANN results for all test samples, Table S6: Gaussian model parameters.
