# Supplementary material for: Neural Network Modelling of Track Profile in Cold Spray Additive Manufacturing
Source: Materials (Basel). 2019 Sep 2;12(17):2827. doi: 10.3390/ma12172827 (PMC6747964; doi:10.3390/ma12172827)
Supplement: Supplementary file 1 [file materials-12-02827-s001.pdf]

materials

Supplementary Materials

Neural Network Modelling of Track Profile in Cold Spray Additive Manufacturing

Daiki Ikeuchi [1,2,*], Alejandro Vargas-Uscategui [2], Xiaofeng Wu [1] and Peter C. King [2]

[1] School of Aerospace, Mechanical and Mechatronic Engineering, The University of Sydney, Sydney, 2006, NSW, Australia
[2] Commonwealth Scientific and Industrial Research Organisation Manufacturing, Private Bag 10, Clayton, 3169, VIC, Australia
* Correspondence: daiki.ikeuchi@sydney.edu.au

Experimental design matrix of input parameters in the training dataset

Table S1 shows the experiment design matrix of input parameters in the training dataset for the ANN model presented in the main body. The dataset was defined by input parameter selection and prepared using a full factorial approach, requiring 36 sample fabrications with a mixture of four-level spray angle (45°, 60°, 75°, 90°), three-level traverse speed (25 mm/s, 100 mm/s and 200 mm/s) and three-level standoff distances (30 mm, 40 mm and 50 mm). The run order indicates the order of fabrication which was randomly selected as discussed in the main body.

Table S1. Input parameters in the training dataset.

Sample ID	Run Order	Spray Angle (°)	Traverse Speed (mm/s)	Standoff Distance (mm)
1	6	45	25	30
2	48	60	25	30
3	1	75	25	30
4	35	90	25	30
5	38	45	100	30
6	40	60	100	30
7	4	75	100	30
8	24	90	100	30
9	18	45	200	30
10	37	60	200	30
11	28	75	200	30
12	44	90	200	30
13	13	45	25	40
14	19	60	25	40
15	12	75	25	40
16	10	90	25	40
17	41	45	100	40
18	33	60	100	40
19	32	75	100	40
20	11	90	100	40
21	39	45	200	40
22	34	60	200	40
23	21	75	200	40
24	31	90	200	40
25	25	45	25	50
26	3	60	25	50

27	15	75	25	50
28	8	90	25	50
29	14	45	100	50
30	16	60	100	50
31	27	75	100	50
32	2	90	100	50
33	43	45	200	50
34	46	60	200	50
35	30	75	200	50
36	29	90	200	50

Experimental design matrix of input parameters in the testing dataset

Table S2 shows the experiment design matrix of input parameters in the testing dataset for the ANN model presented in the main body. The dataset was defined by the 75-25 data division rule, requiring 12 sample fabrications with randomly selected parameters values between their minimum and maximum: spray angle (45° and 90°), traverse speed (25 mm/s and 200 mm/s) and standoff distance (30 mm and 50 mm) with the aid of MATLAB version R2018a.

Table S2. Input parameters in the testing dataset.

Sample ID	Run Order	Spray Angle (°)	Traverse Speed (mm/s)	Standoff Distance (mm)
37	20	86	75	45
38	42	79	92	41
39	22	48	34	41
40	7	80	188	33
41	36	71	107	30
42	23	60	53	46
43	17	59	117	33
44	26	72	71	43
45	5	76	156	39
46	9	49	65	48
47	45	52	170	41
48	47	90	39	39

Table S3. Output parameters in the training dataset.

-	Sample ID											
**Output**	**1**	**2**	**3**	**4**	**5**	**6**	**7**	**8**	**9**	**10**	**11**	**12**
1	5.074	4.410	3.815	3.783	4.166	3.931	3.619	3.583	3.571	3.081	3.002	2.996
2	3.516	3.267	3.098	2.994	2.213	2.208	2.291	2.266	1.513	1.627	1.706	1.465
3	2.823	2.725	2.651	2.570	1.641	1.643	1.687	1.749	1.013	1.194	1.226	1.087
4	2.404	2.383	2.363	2.305	1.339	1.348	1.473	1.426	0.684	0.917	0.975	0.811
5	2.103	2.128	2.155	2.100	1.129	1.166	1.232	1.257	0.568	0.760	0.808	0.712
6	1.932	1.943	1.996	1.945	0.942	1.005	1.112	1.136	0.510	0.616	0.690	0.589
7	1.734	1.815	1.873	1.832	0.824	0.864	1.026	1.016	0.399	0.540	0.592	0.521
8	1.622	1.708	1.773	1.743	0.732	0.796	0.918	0.913	0.361	0.488	0.521	0.471
9	1.470	1.620	1.691	1.671	0.655	0.739	0.860	0.844	0.319	0.439	0.468	0.429
10	1.434	1.543	1.624	1.612	0.596	0.686	0.781	0.796	0.280	0.394	0.424	0.385
11	1.356	1.470	1.567	1.561	0.539	0.624	0.731	0.745	0.252	0.359	0.386	0.342
12	1.281	1.408	1.518	1.520	0.496	0.577	0.690	0.695	0.225	0.333	0.357	0.315
13	1.230	1.356	1.480	1.484	0.461	0.545	0.653	0.651	0.202	0.311	0.334	0.293
14	1.171	1.310	1.444	1.456	0.434	0.530	0.625	0.615	0.189	0.291	0.314	0.274
15	1.118	1.271	1.415	1.434	0.413	0.514	0.584	0.587	0.180	0.275	0.296	0.259
16	1.082	1.237	1.388	1.416	0.394	0.490	0.555	0.558	0.172	0.261	0.280	0.248

17	1.067	1.208	1.368	1.404	0.374	0.463	0.539	0.532	0.165	0.249	0.266	0.238
18	1.061	1.184	1.350	1.394	0.358	0.439	0.522	0.512	0.158	0.239	0.254	0.228
19	1.051	1.164	1.338	1.390	0.345	0.421	0.502	0.494	0.151	0.231	0.244	0.220
20	1.036	1.149	1.327	1.385	0.334	0.406	0.482	0.478	0.145	0.223	0.235	0.213
21	1.016	1.134	1.322	1.386	0.324	0.398	0.468	0.466	0.140	0.216	0.227	0.207
22	0.984	1.125	1.319	1.389	0.315	0.393	0.456	0.455	0.135	0.209	0.220	0.203
23	0.941	1.118	1.319	1.394	0.306	0.387	0.444	0.443	0.131	0.204	0.214	0.199
24	0.922	1.114	1.322	1.403	0.299	0.375	0.434	0.432	0.126	0.199	0.208	0.196
25	0.926	1.113	1.326	1.411	0.292	0.365	0.422	0.425	0.122	0.195	0.203	0.194
26	0.935	1.112	1.334	1.424	0.287	0.358	0.413	0.419	0.118	0.192	0.199	0.191
27	0.940	1.114	1.342	1.435	0.282	0.350	0.406	0.414	0.114	0.189	0.196	0.189
28	0.934	1.117	1.355	1.451	0.278	0.341	0.397	0.411	0.111	0.187	0.192	0.188
29	0.931	1.122	1.368	1.465	0.275	0.332	0.391	0.407	0.108	0.185	0.190	0.186
30	0.934	1.130	1.385	1.481	0.272	0.327	0.388	0.404	0.106	0.183	0.187	0.185
31	0.929	1.138	1.403	1.495	0.271	0.324	0.387	0.400	0.105	0.182	0.185	0.184
32	0.929	1.150	1.424	1.510	0.270	0.324	0.387	0.397	0.104	0.181	0.183	0.182
33	0.944	1.163	1.445	1.522	0.269	0.324	0.389	0.395	0.104	0.181	0.182	0.181
34	0.966	1.179	1.468	1.534	0.269	0.325	0.388	0.394	0.105	0.181	0.181	0.180
35	0.972	1.198	1.488	1.542	0.270	0.325	0.386	0.394	0.105	0.181	0.180	0.180
36	0.970	1.220	1.508	1.548	0.271	0.323	0.388	0.395	0.107	0.182	0.180	0.180
37	0.984	1.246	1.525	1.552	0.273	0.322	0.390	0.397	0.108	0.183	0.180	0.181
38	1.018	1.272	1.540	1.552	0.276	0.323	0.392	0.400	0.110	0.184	0.180	0.182
39	1.053	1.300	1.553	1.548	0.280	0.325	0.394	0.404	0.113	0.186	0.180	0.184
40	1.087	1.330	1.564	1.542	0.285	0.330	0.398	0.408	0.116	0.188	0.181	0.186
41	1.103	1.360	1.571	1.535	0.290	0.338	0.404	0.414	0.119	0.190	0.183	0.189
42	1.146	1.391	1.577	1.527	0.296	0.349	0.412	0.419	0.123	0.193	0.185	0.193
43	1.191	1.423	1.581	1.520	0.302	0.362	0.416	0.426	0.127	0.195	0.187	0.197
44	1.230	1.456	1.583	1.513	0.308	0.374	0.419	0.433	0.132	0.199	0.190	0.203
45	1.278	1.490	1.584	1.509	0.316	0.387	0.425	0.440	0.136	0.202	0.194	0.209
46	1.330	1.523	1.583	1.506	0.323	0.398	0.429	0.449	0.141	0.207	0.199	0.216
47	1.404	1.555	1.582	1.505	0.331	0.411	0.433	0.460	0.146	0.212	0.204	0.224
48	1.445	1.584	1.582	1.507	0.340	0.426	0.440	0.473	0.153	0.218	0.211	0.233
49	1.517	1.611	1.583	1.510	0.351	0.442	0.454	0.489	0.160	0.226	0.219	0.242
50	1.594	1.638	1.586	1.517	0.364	0.453	0.481	0.509	0.168	0.234	0.228	0.253
51	1.673	1.664	1.591	1.525	0.381	0.475	0.509	0.532	0.176	0.244	0.239	0.265
52	1.731	1.688	1.602	1.539	0.398	0.508	0.529	0.552	0.185	0.257	0.252	0.279
53	1.785	1.716	1.616	1.555	0.416	0.536	0.546	0.573	0.194	0.271	0.268	0.298
54	1.853	1.742	1.633	1.579	0.440	0.564	0.573	0.597	0.205	0.287	0.285	0.319
55	1.929	1.771	1.656	1.606	0.471	0.595	0.616	0.627	0.220	0.308	0.305	0.344
56	1.982	1.801	1.684	1.643	0.513	0.632	0.645	0.660	0.240	0.333	0.328	0.371
57	2.048	1.837	1.719	1.686	0.558	0.690	0.673	0.698	0.260	0.362	0.356	0.399
58	2.108	1.882	1.761	1.737	0.612	0.750	0.712	0.752	0.282	0.402	0.389	0.437
59	2.175	1.932	1.811	1.801	0.687	0.816	0.779	0.804	0.312	0.451	0.427	0.488
60	2.274	1.998	1.876	1.876	0.767	0.906	0.813	0.872	0.355	0.505	0.469	0.549
61	2.376	2.086	1.957	1.965	0.860	0.981	0.924	0.957	0.395	0.566	0.523	0.613
62	2.466	2.200	2.064	2.075	0.952	1.086	1.027	1.064	0.449	0.639	0.605	0.702
63	2.610	2.350	2.203	2.217	1.082	1.261	1.161	1.195	0.581	0.765	0.710	0.846
64	2.826	2.553	2.383	2.408	1.329	1.448	1.327	1.356	0.747	0.917	0.835	1.014
65	3.107	2.848	2.629	2.678	1.578	1.771	1.561	1.689	1.067	1.181	1.052	1.307
66	3.651	3.357	3.052	3.094	2.167	2.272	1.970	2.101	1.386	1.599	1.489	1.786
67	5.074	4.410	3.815	3.783	4.166	3.931	3.619	3.583	3.571	3.081	3.002	2.996

Table S3. continued. Output parameters in the training dataset.

-	Sample ID											
**Output**	**13**	**14**	**15**	**16**	**17**	**18**	**19**	**20**	**21**	**22**	**23**	**24**
1	5.121	4.465	3.940	3.878	4.173	3.900	3.606	3.603	3.624	3.418	3.128	3.039
2	3.700	3.396	3.139	3.160	2.318	2.200	2.332	2.387	1.538	1.767	1.795	1.780

3	2.905	2.850	2.662	2.717	1.592	1.687	1.699	1.784	0.996	1.336	1.212	1.246
4	2.424	2.431	2.369	2.430	1.257	1.351	1.448	1.444	0.726	0.925	0.934	0.998
5	2.159	2.228	2.150	2.213	1.031	1.148	1.255	1.250	0.514	0.755	0.756	0.845
6	1.945	1.997	1.987	2.060	0.924	1.004	1.093	1.105	0.416	0.628	0.653	0.686
7	1.795	1.823	1.858	1.941	0.792	0.906	0.980	1.009	0.377	0.545	0.570	0.537
8	1.636	1.740	1.755	1.845	0.703	0.811	0.901	0.891	0.347	0.500	0.503	0.494
9	1.555	1.601	1.671	1.763	0.655	0.737	0.823	0.829	0.318	0.446	0.451	0.462
10	1.448	1.557	1.600	1.696	0.592	0.687	0.759	0.754	0.285	0.394	0.401	0.431
11	1.399	1.492	1.539	1.642	0.543	0.648	0.692	0.709	0.250	0.349	0.373	0.390
12	1.322	1.402	1.489	1.597	0.500	0.609	0.637	0.666	0.226	0.318	0.330	0.352
13	1.249	1.358	1.448	1.558	0.459	0.563	0.602	0.638	0.209	0.292	0.313	0.330
14	1.219	1.309	1.413	1.529	0.432	0.525	0.578	0.598	0.194	0.274	0.292	0.308
15	1.157	1.268	1.384	1.502	0.413	0.498	0.554	0.555	0.181	0.256	0.275	0.284
16	1.093	1.251	1.358	1.483	0.394	0.474	0.532	0.526	0.170	0.242	0.262	0.272
17	1.065	1.238	1.338	1.465	0.379	0.452	0.507	0.509	0.162	0.232	0.248	0.262
18	1.015	1.199	1.321	1.453	0.365	0.433	0.491	0.497	0.156	0.224	0.235	0.252
19	0.994	1.189	1.307	1.442	0.350	0.417	0.477	0.482	0.150	0.216	0.226	0.238
20	0.972	1.163	1.296	1.437	0.336	0.404	0.464	0.460	0.145	0.209	0.218	0.223
21	0.957	1.147	1.286	1.433	0.325	0.392	0.447	0.441	0.140	0.202	0.213	0.213
22	0.955	1.135	1.280	1.433	0.316	0.382	0.430	0.425	0.136	0.197	0.209	0.207
23	0.947	1.119	1.275	1.435	0.307	0.372	0.422	0.409	0.132	0.193	0.206	0.203
24	0.936	1.100	1.275	1.439	0.300	0.363	0.413	0.398	0.128	0.190	0.204	0.200
25	0.926	1.106	1.277	1.445	0.294	0.355	0.403	0.391	0.125	0.187	0.201	0.198
26	0.917	1.108	1.282	1.451	0.289	0.348	0.396	0.384	0.122	0.185	0.197	0.196
27	0.903	1.091	1.290	1.459	0.284	0.343	0.391	0.379	0.119	0.183	0.192	0.194
28	0.898	1.109	1.300	1.466	0.280	0.338	0.384	0.378	0.117	0.181	0.187	0.191
29	0.904	1.116	1.315	1.475	0.276	0.333	0.380	0.376	0.115	0.178	0.183	0.189
30	0.906	1.109	1.331	1.484	0.271	0.329	0.377	0.372	0.113	0.176	0.179	0.186
31	0.894	1.116	1.351	1.492	0.265	0.326	0.377	0.365	0.112	0.175	0.176	0.183
32	0.893	1.119	1.370	1.500	0.260	0.325	0.379	0.360	0.111	0.174	0.174	0.181
33	0.898	1.110	1.391	1.506	0.256	0.325	0.375	0.355	0.111	0.173	0.172	0.179
34	0.900	1.121	1.410	1.509	0.253	0.325	0.375	0.351	0.111	0.172	0.171	0.179
35	0.918	1.131	1.430	1.508	0.250	0.327	0.377	0.350	0.111	0.172	0.170	0.180
36	0.938	1.135	1.448	1.504	0.251	0.329	0.381	0.350	0.112	0.172	0.170	0.180
37	0.963	1.166	1.465	1.499	0.254	0.332	0.382	0.353	0.113	0.173	0.170	0.181
38	0.992	1.195	1.481	1.492	0.259	0.335	0.382	0.355	0.115	0.174	0.171	0.182
39	1.011	1.237	1.495	1.484	0.266	0.339	0.383	0.358	0.116	0.174	0.172	0.184
40	1.015	1.271	1.508	1.477	0.275	0.345	0.386	0.360	0.118	0.175	0.173	0.185
41	1.030	1.307	1.520	1.468	0.285	0.352	0.392	0.365	0.121	0.175	0.174	0.187
42	1.068	1.344	1.527	1.461	0.293	0.359	0.399	0.369	0.123	0.176	0.176	0.190
43	1.105	1.374	1.535	1.454	0.300	0.367	0.406	0.374	0.126	0.177	0.179	0.194
44	1.156	1.413	1.539	1.449	0.307	0.376	0.414	0.380	0.128	0.179	0.182	0.199
45	1.182	1.457	1.544	1.445	0.315	0.387	0.427	0.389	0.131	0.181	0.185	0.206
46	1.205	1.505	1.547	1.444	0.323	0.400	0.443	0.397	0.135	0.184	0.190	0.213
47	1.259	1.557	1.550	1.444	0.331	0.415	0.458	0.404	0.139	0.188	0.197	0.221
48	1.290	1.601	1.554	1.447	0.340	0.430	0.471	0.413	0.144	0.192	0.206	0.231
49	1.359	1.640	1.560	1.454	0.352	0.446	0.487	0.423	0.149	0.197	0.218	0.240
50	1.410	1.686	1.568	1.462	0.367	0.465	0.507	0.444	0.155	0.203	0.231	0.252
51	1.453	1.728	1.579	1.475	0.387	0.488	0.530	0.466	0.163	0.211	0.243	0.265
52	1.506	1.762	1.593	1.489	0.411	0.515	0.547	0.488	0.171	0.223	0.255	0.279
53	1.574	1.804	1.611	1.509	0.433	0.544	0.571	0.507	0.178	0.243	0.267	0.290
54	1.625	1.838	1.632	1.531	0.450	0.577	0.602	0.527	0.186	0.273	0.283	0.302
55	1.711	1.879	1.661	1.563	0.473	0.612	0.629	0.553	0.196	0.300	0.300	0.318
56	1.766	1.916	1.693	1.601	0.500	0.648	0.665	0.585	0.209	0.324	0.321	0.346
57	1.835	1.968	1.731	1.644	0.550	0.690	0.709	0.617	0.228	0.349	0.345	0.383
58	1.887	2.017	1.776	1.695	0.607	0.744	0.774	0.656	0.254	0.375	0.368	0.419
59	1.961	2.076	1.833	1.758	0.678	0.806	0.823	0.704	0.279	0.412	0.407	0.448
60	2.077	2.140	1.902	1.834	0.743	0.887	0.892	0.776	0.307	0.458	0.458	0.500

61	2.198	2.231	1.987	1.927	0.812	1.000	1.005	0.870	0.354	0.525	0.515	0.573
62	2.336	2.336	2.094	2.046	0.975	1.100	1.106	0.963	0.413	0.643	0.605	0.662
63	2.489	2.489	2.232	2.192	1.150	1.236	1.276	1.084	0.547	0.730	0.683	0.794
64	2.711	2.700	2.423	2.387	1.289	1.468	1.489	1.301	0.630	0.929	0.853	0.881
65	3.076	3.041	2.716	2.686	1.620	1.741	1.724	1.520	0.848	1.145	1.049	1.198
66	3.733	3.538	3.145	3.129	2.284	2.279	2.340	2.082	1.323	1.592	1.470	1.604
67	5.121	4.465	3.940	3.878	4.173	3.900	3.606	3.603	3.624	3.418	3.128	3.039

Table S3. continued. Output parameters in the training dataset.

-	Sample ID											
**Output**	**25**	**26**	**27**	**28**	**29**	**30**	**31**	**32**	**33**	**34**	**35**	**36**
1	5.152	4.486	4.160	4.098	4.591	3.753	3.724	3.673	3.697	3.506	3.438	3.086
2	3.588	3.526	3.140	3.219	2.334	2.215	2.185	2.143	1.822	1.581	1.713	1.809
3	2.883	2.853	2.603	2.706	1.709	1.620	1.689	1.693	1.099	1.061	1.237	1.220
4	2.487	2.457	2.300	2.405	1.299	1.332	1.433	1.427	0.779	0.845	0.975	0.966
5	2.161	2.146	2.080	2.196	1.102	1.069	1.150	1.190	0.619	0.716	0.790	0.795
6	1.922	1.989	1.921	2.047	0.968	0.996	1.011	1.088	0.464	0.608	0.654	0.683
7	1.750	1.779	1.797	1.928	0.813	0.836	0.915	1.006	0.417	0.515	0.547	0.556
8	1.576	1.684	1.694	1.827	0.742	0.789	0.824	0.889	0.364	0.467	0.473	0.498
9	1.509	1.607	1.607	1.745	0.688	0.722	0.725	0.790	0.315	0.411	0.426	0.461
10	1.419	1.520	1.534	1.677	0.592	0.636	0.678	0.758	0.290	0.366	0.383	0.417
11	1.350	1.446	1.474	1.619	0.542	0.612	0.648	0.719	0.265	0.324	0.347	0.381
12	1.249	1.367	1.422	1.570	0.500	0.568	0.605	0.648	0.243	0.297	0.320	0.351
13	1.192	1.312	1.376	1.531	0.489	0.516	0.566	0.614	0.220	0.272	0.298	0.330
14	1.143	1.276	1.339	1.497	0.473	0.483	0.540	0.581	0.202	0.253	0.280	0.311
15	1.094	1.249	1.303	1.470	0.431	0.462	0.508	0.552	0.188	0.241	0.264	0.292
16	1.053	1.215	1.274	1.444	0.411	0.443	0.477	0.533	0.177	0.232	0.250	0.274
17	1.018	1.177	1.247	1.426	0.391	0.425	0.461	0.515	0.167	0.223	0.238	0.260
18	0.984	1.163	1.227	1.409	0.374	0.408	0.434	0.488	0.159	0.216	0.227	0.248
19	0.959	1.140	1.209	1.398	0.359	0.390	0.411	0.471	0.151	0.207	0.218	0.237
20	0.942	1.117	1.195	1.388	0.346	0.380	0.401	0.460	0.145	0.200	0.210	0.226
21	0.923	1.103	1.184	1.382	0.334	0.371	0.395	0.446	0.139	0.194	0.204	0.216
22	0.906	1.090	1.175	1.377	0.322	0.362	0.388	0.431	0.135	0.187	0.198	0.208
23	0.890	1.091	1.171	1.376	0.312	0.356	0.375	0.419	0.131	0.181	0.192	0.201
24	0.881	1.090	1.168	1.377	0.298	0.345	0.364	0.409	0.127	0.177	0.188	0.196
25	0.872	1.078	1.170	1.379	0.285	0.331	0.357	0.399	0.124	0.173	0.184	0.191
26	0.867	1.070	1.172	1.383	0.275	0.323	0.350	0.390	0.122	0.169	0.180	0.187
27	0.861	1.068	1.177	1.388	0.268	0.317	0.346	0.378	0.120	0.166	0.177	0.184
28	0.856	1.068	1.184	1.394	0.262	0.313	0.340	0.369	0.118	0.163	0.175	0.181
29	0.853	1.062	1.193	1.400	0.258	0.310	0.334	0.363	0.117	0.161	0.173	0.179
30	0.851	1.056	1.205	1.407	0.257	0.308	0.331	0.358	0.116	0.159	0.171	0.177
31	0.854	1.047	1.218	1.414	0.258	0.308	0.332	0.356	0.116	0.157	0.169	0.175
32	0.863	1.041	1.233	1.421	0.259	0.311	0.333	0.356	0.115	0.155	0.168	0.174
33	0.878	1.040	1.250	1.427	0.259	0.317	0.334	0.356	0.115	0.154	0.167	0.174
34	0.891	1.045	1.268	1.432	0.259	0.323	0.336	0.357	0.116	0.153	0.166	0.174
35	0.905	1.046	1.287	1.434	0.258	0.322	0.339	0.360	0.116	0.153	0.165	0.175
36	0.923	1.057	1.307	1.434	0.257	0.319	0.340	0.363	0.117	0.152	0.166	0.177
37	0.946	1.072	1.327	1.431	0.257	0.319	0.342	0.365	0.118	0.152	0.166	0.179
38	0.968	1.090	1.346	1.427	0.259	0.322	0.345	0.365	0.119	0.153	0.167	0.181
39	0.981	1.114	1.365	1.421	0.262	0.329	0.347	0.366	0.120	0.154	0.169	0.182
40	0.991	1.144	1.383	1.415	0.266	0.340	0.347	0.369	0.121	0.157	0.171	0.184
41	1.008	1.200	1.400	1.410	0.271	0.349	0.348	0.374	0.123	0.160	0.173	0.187
42	1.027	1.245	1.415	1.407	0.277	0.357	0.352	0.380	0.125	0.164	0.176	0.189
43	1.047	1.279	1.431	1.403	0.282	0.367	0.359	0.388	0.127	0.169	0.180	0.192
44	1.075	1.305	1.443	1.403	0.288	0.376	0.368	0.397	0.129	0.176	0.184	0.196
45	1.121	1.357	1.456	1.404	0.296	0.382	0.378	0.406	0.132	0.184	0.189	0.200
46	1.175	1.407	1.468	1.407	0.305	0.385	0.386	0.416	0.134	0.191	0.194	0.206

47	1.227	1.463	1.482	1.411	0.316	0.390	0.395	0.423	0.136	0.199	0.199	0.213
48	1.273	1.509	1.495	1.418	0.330	0.394	0.410	0.431	0.140	0.206	0.206	0.223
49	1.327	1.552	1.511	1.427	0.348	0.408	0.432	0.441	0.145	0.212	0.213	0.233
50	1.386	1.608	1.527	1.439	0.364	0.436	0.449	0.455	0.151	0.220	0.221	0.245
51	1.449	1.633	1.547	1.456	0.386	0.462	0.469	0.473	0.159	0.228	0.230	0.256
52	1.519	1.667	1.569	1.474	0.411	0.497	0.507	0.502	0.168	0.239	0.241	0.267
53	1.597	1.707	1.595	1.499	0.440	0.523	0.528	0.532	0.177	0.254	0.253	0.278
54	1.671	1.756	1.626	1.527	0.460	0.553	0.550	0.557	0.187	0.272	0.267	0.296
55	1.754	1.790	1.660	1.562	0.475	0.580	0.585	0.592	0.199	0.294	0.283	0.318
56	1.827	1.835	1.702	1.603	0.493	0.612	0.628	0.628	0.212	0.313	0.303	0.347
57	1.886	1.896	1.748	1.650	0.523	0.666	0.655	0.680	0.229	0.330	0.326	0.383
58	1.962	1.949	1.802	1.710	0.571	0.722	0.714	0.725	0.259	0.352	0.357	0.443
59	2.058	2.013	1.868	1.779	0.624	0.802	0.776	0.777	0.305	0.386	0.399	0.474
60	2.172	2.088	1.946	1.861	0.721	0.857	0.870	0.852	0.346	0.445	0.453	0.517
61	2.284	2.173	2.044	1.962	0.830	0.950	0.947	0.917	0.389	0.545	0.511	0.585
62	2.419	2.284	2.169	2.087	0.926	1.080	1.054	1.001	0.447	0.628	0.576	0.675
63	2.605	2.456	2.326	2.246	1.054	1.194	1.211	1.182	0.604	0.748	0.684	0.767
64	2.813	2.669	2.533	2.455	1.297	1.359	1.380	1.374	0.708	0.891	0.858	0.936
65	3.216	2.986	2.837	2.757	1.700	1.727	1.668	1.571	0.980	1.194	1.139	1.224
66	3.783	3.441	3.306	3.216	2.215	2.250	2.161	2.060	1.412	1.841	1.585	1.743
67	5.152	4.486	4.160	4.098	4.591	3.753	3.724	3.673	3.697	3.506	3.438	3.086

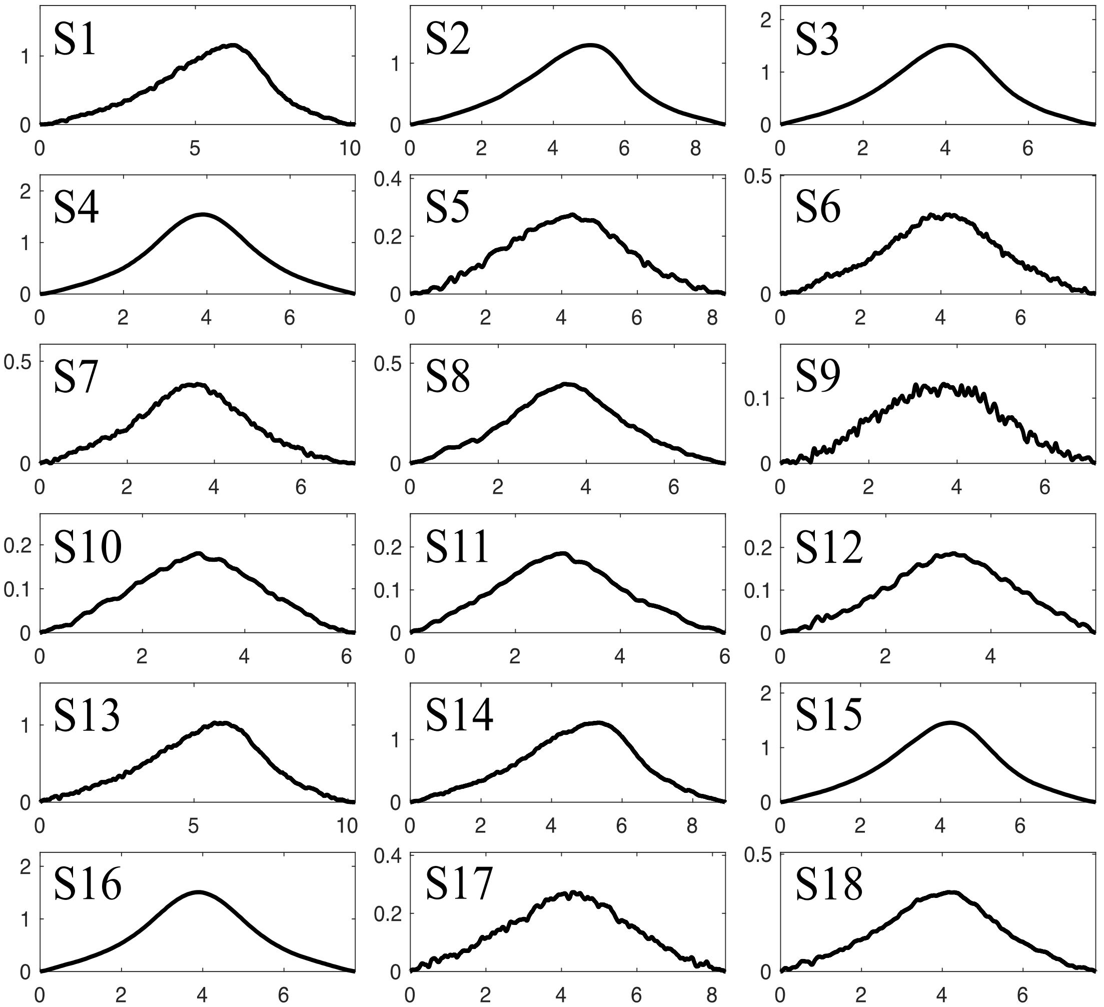

Figure S1. The measured track profiles of Sample 1 to Sample 18. The vertical axis is Deposit height (mm), while the horizontal axis is the Horizontal location on substrate (mm).

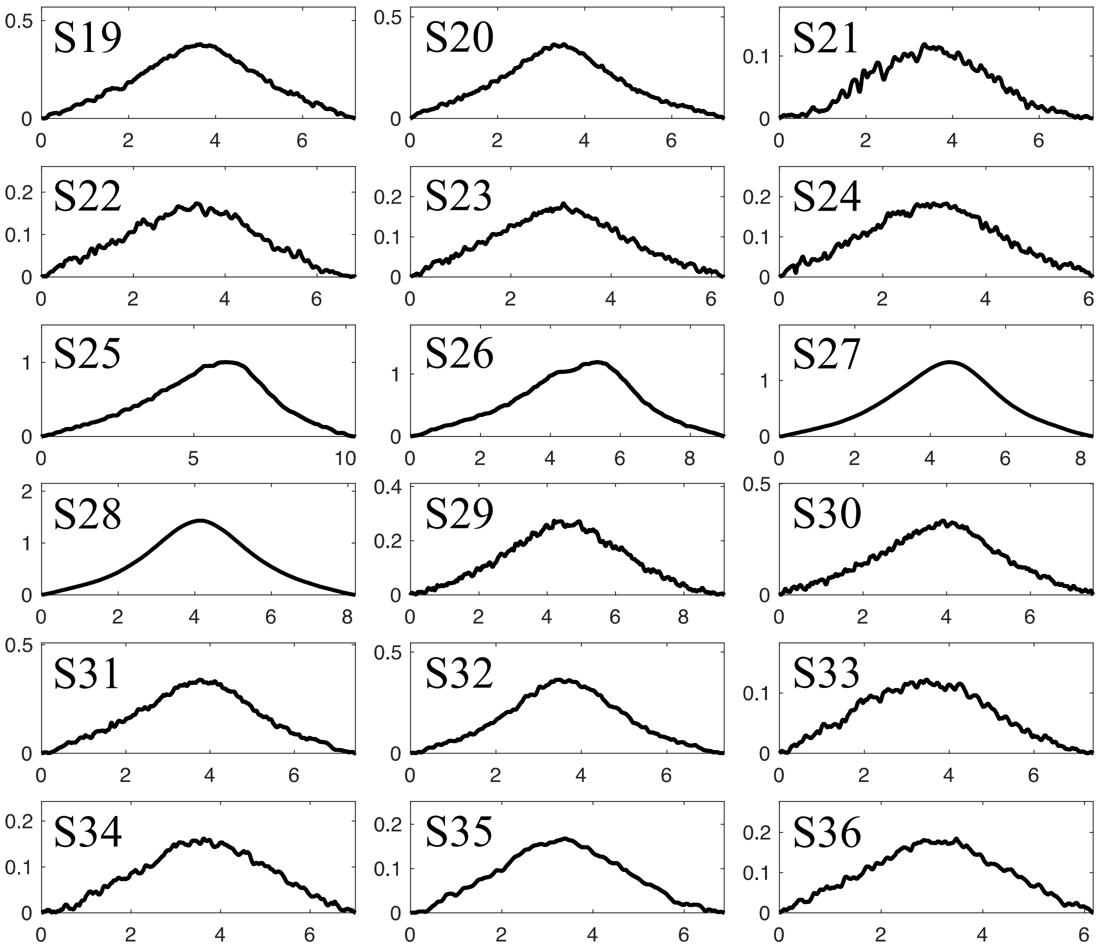

Figure S2. The measured track profiles of Sample 19 to Sample 36. The vertical axis is Deposit height (mm), while the horizontal axis is the Horizontal location on substrate (mm).

Output parameters in the testing dataset

Table S4 shows the output parameter values for each testing sample (i.e., 67 parameters for each taken using the polar coordinate method discussed and verified in the main body). The output parameters are polar lengths in mm. Sample 37 to Sample 48 are in the testing dataset. These output parameter values are taken from the measured track profiles as described in the main body and presented in Figure S3.

Table S4. Output parameters in the testing dataset.

-	Sample ID											
**Output**	**37**	**38**	**39**	**40**	**41**	**42**	**43**	**44**	**45**	**46**	**47**	**48**
1	3.948	3.901	4.995	3.115	3.529	4.268	3.701	4.018	3.133	4.901	3.724	4.103
2	2.581	2.358	3.270	1.805	2.127	2.703	2.058	2.563	1.828	2.813	1.709	3.052
3	2.002	1.702	2.486	1.281	1.641	2.144	1.567	2.064	1.310	2.013	1.191	2.556
4	1.693	1.408	2.120	1.025	1.320	1.803	1.308	1.677	1.054	1.687	0.919	2.281
5	1.509	1.185	1.861	0.833	1.153	1.601	1.047	1.480	0.907	1.443	0.707	1.974
6	1.333	1.056	1.616	0.714	1.000	1.383	0.908	1.362	0.784	1.267	0.589	1.794
7	1.217	0.931	1.533	0.620	0.904	1.252	0.829	1.240	0.680	1.097	0.522	1.673
8	1.135	0.855	1.379	0.554	0.791	1.177	0.724	1.117	0.602	1.015	0.419	1.584
9	1.029	0.746	1.291	0.522	0.735	1.083	0.646	1.032	0.539	0.926	0.377	1.499
10	0.956	0.700	1.223	0.477	0.696	1.008	0.590	0.984	0.470	0.864	0.350	1.415
11	0.915	0.655	1.152	0.430	0.645	0.955	0.559	0.902	0.426	0.805	0.325	1.345
12	0.869	0.604	1.069	0.404	0.605	0.904	0.519	0.849	0.400	0.755	0.301	1.292
13	0.826	0.569	0.997	0.375	0.579	0.857	0.485	0.805	0.383	0.701	0.268	1.258

14	0.781	0.539	0.961	0.344	0.528	0.808	0.462	0.760	0.367	0.660	0.243	1.222
15	0.761	0.510	0.926	0.323	0.495	0.773	0.446	0.715	0.352	0.625	0.223	1.175
16	0.731	0.491	0.896	0.307	0.481	0.743	0.431	0.690	0.337	0.592	0.208	1.129
17	0.702	0.455	0.874	0.293	0.465	0.724	0.420	0.668	0.323	0.566	0.196	1.092
18	0.680	0.435	0.850	0.280	0.448	0.711	0.408	0.643	0.310	0.541	0.186	1.069
19	0.662	0.416	0.828	0.269	0.424	0.684	0.391	0.625	0.296	0.519	0.178	1.051
20	0.634	0.406	0.813	0.258	0.404	0.649	0.365	0.605	0.281	0.503	0.172	1.022
21	0.614	0.401	0.791	0.247	0.387	0.636	0.351	0.584	0.265	0.496	0.166	1.009
22	0.601	0.394	0.773	0.236	0.378	0.632	0.347	0.562	0.253	0.489	0.161	0.996
23	0.588	0.383	0.764	0.228	0.371	0.624	0.345	0.552	0.246	0.479	0.157	0.996
24	0.575	0.369	0.754	0.222	0.367	0.604	0.338	0.541	0.241	0.467	0.153	0.988
25	0.566	0.358	0.744	0.217	0.362	0.586	0.326	0.527	0.237	0.457	0.151	0.994
26	0.556	0.348	0.739	0.214	0.354	0.577	0.312	0.519	0.234	0.448	0.148	0.982
27	0.545	0.341	0.738	0.211	0.341	0.576	0.303	0.514	0.231	0.438	0.146	0.980
28	0.538	0.338	0.731	0.210	0.333	0.577	0.295	0.507	0.229	0.425	0.145	0.977
29	0.534	0.339	0.722	0.209	0.330	0.577	0.290	0.501	0.227	0.414	0.144	0.964
30	0.532	0.341	0.714	0.208	0.329	0.575	0.288	0.498	0.226	0.411	0.143	0.967
31	0.530	0.342	0.714	0.207	0.330	0.570	0.288	0.494	0.226	0.415	0.142	0.978
32	0.526	0.339	0.721	0.205	0.332	0.565	0.289	0.485	0.227	0.422	0.142	0.970
33	0.521	0.335	0.732	0.203	0.333	0.570	0.292	0.479	0.228	0.428	0.141	0.960
34	0.514	0.330	0.743	0.201	0.334	0.579	0.297	0.480	0.228	0.430	0.142	0.961
35	0.511	0.326	0.753	0.200	0.336	0.576	0.304	0.482	0.228	0.430	0.142	0.964
36	0.514	0.323	0.763	0.199	0.340	0.574	0.310	0.483	0.227	0.431	0.143	0.960
37	0.522	0.323	0.777	0.200	0.343	0.576	0.312	0.485	0.227	0.436	0.144	0.964
38	0.525	0.323	0.792	0.201	0.346	0.578	0.312	0.487	0.227	0.443	0.146	0.969
39	0.529	0.325	0.807	0.202	0.348	0.581	0.312	0.489	0.228	0.451	0.148	0.971
40	0.536	0.330	0.820	0.204	0.352	0.591	0.314	0.499	0.231	0.459	0.151	0.971
41	0.546	0.337	0.830	0.206	0.358	0.604	0.318	0.510	0.236	0.470	0.154	0.976
42	0.556	0.346	0.844	0.209	0.361	0.613	0.323	0.515	0.242	0.480	0.157	0.980
43	0.566	0.357	0.869	0.213	0.366	0.621	0.331	0.519	0.249	0.488	0.161	1.001
44	0.572	0.367	0.897	0.219	0.373	0.634	0.339	0.529	0.255	0.494	0.164	1.015
45	0.581	0.374	0.920	0.226	0.385	0.671	0.350	0.546	0.260	0.500	0.168	1.024
46	0.592	0.381	0.945	0.234	0.396	0.694	0.364	0.555	0.267	0.510	0.172	1.038
47	0.607	0.390	0.989	0.242	0.405	0.708	0.379	0.564	0.275	0.537	0.177	1.051
48	0.631	0.401	1.048	0.252	0.419	0.729	0.396	0.574	0.283	0.570	0.182	1.059
49	0.648	0.419	1.102	0.264	0.439	0.750	0.413	0.591	0.292	0.594	0.188	1.072
50	0.664	0.432	1.143	0.275	0.461	0.764	0.433	0.610	0.303	0.619	0.194	1.090
51	0.687	0.442	1.185	0.287	0.486	0.778	0.464	0.631	0.319	0.633	0.203	1.111
52	0.704	0.454	1.250	0.297	0.509	0.812	0.482	0.654	0.341	0.651	0.215	1.125
53	0.723	0.477	1.334	0.307	0.528	0.847	0.493	0.680	0.365	0.689	0.232	1.167
54	0.749	0.517	1.396	0.318	0.549	0.883	0.512	0.715	0.392	0.730	0.251	1.195
55	0.787	0.547	1.459	0.336	0.579	0.933	0.545	0.764	0.418	0.774	0.271	1.233
56	0.831	0.583	1.540	0.367	0.619	1.008	0.576	0.807	0.453	0.826	0.291	1.267
57	0.871	0.630	1.607	0.400	0.671	1.062	0.632	0.844	0.488	0.908	0.315	1.308
58	0.924	0.688	1.693	0.431	0.716	1.133	0.693	0.902	0.515	0.976	0.338	1.371
59	0.979	0.745	1.775	0.466	0.779	1.210	0.750	0.940	0.559	1.053	0.369	1.445
60	1.056	0.813	1.898	0.524	0.860	1.295	0.855	1.019	0.626	1.149	0.415	1.518
61	1.159	0.892	2.020	0.604	0.917	1.389	0.941	1.112	0.713	1.258	0.461	1.620
62	1.283	0.999	2.158	0.676	1.065	1.503	1.030	1.234	0.824	1.388	0.541	1.745
63	1.407	1.124	2.314	0.783	1.170	1.690	1.208	1.376	0.942	1.557	0.679	1.888
64	1.644	1.353	2.542	0.927	1.358	1.878	1.428	1.531	1.056	1.775	0.814	2.132
65	1.902	1.647	2.900	1.202	1.621	2.217	1.668	1.838	1.345	2.247	1.045	2.465
66	2.432	2.114	3.567	1.694	2.093	2.735	2.271	2.303	1.815	2.758	1.687	2.978
67	3.948	3.901	4.995	3.115	3.529	4.268	3.701	4.018	3.133	4.901	3.724	4.103

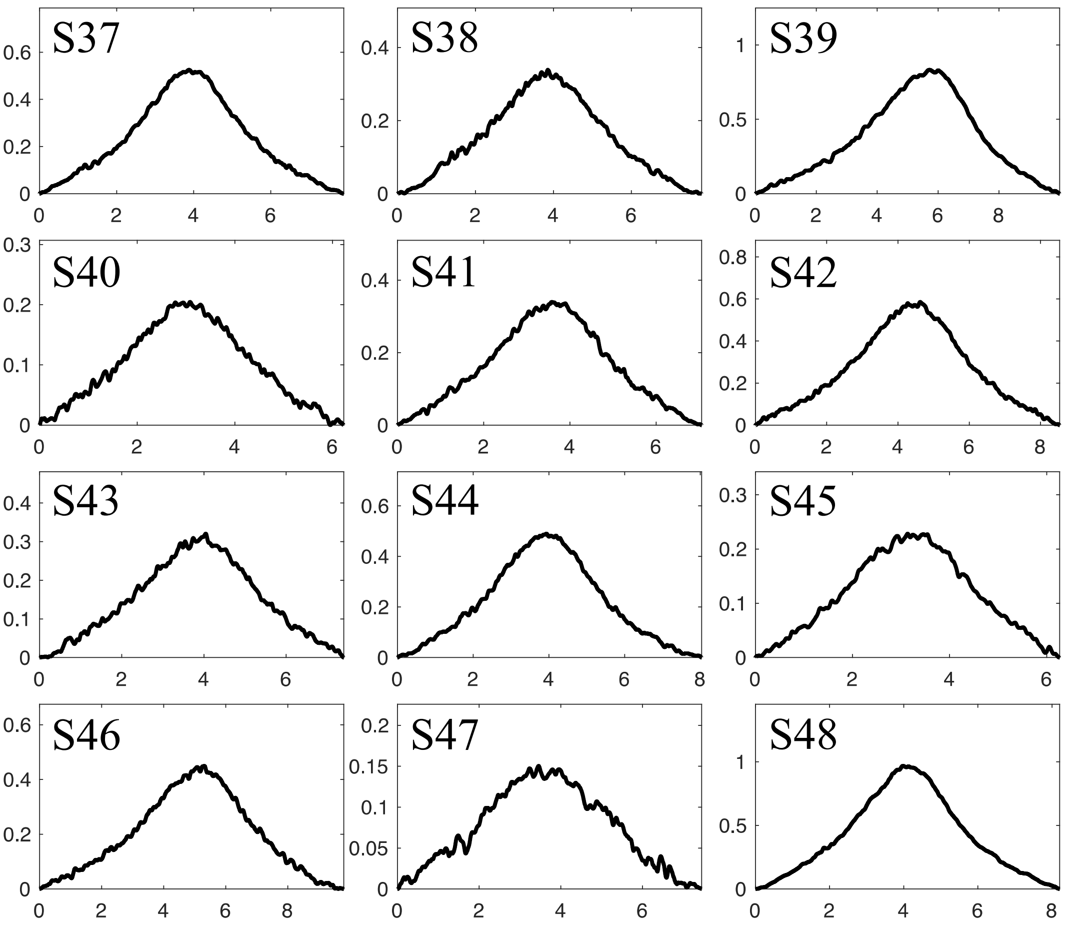

Figure S3. The measured track profiles of Sample 37 to Sample 48. The vertical axis is Deposit height (mm), while the horizontal axis is the Horizontal location on substrate (mm).

ANN results for all test samples

Table S5 lists the prediction results of the developed ANN model for each testing sample. The prediction results represent the polar lengths in mm. Figure S4 shows the predicted track profiles using these ANN predicted results, in comparison to the measured track profiles. Note that Sample 37 and Sample 39 are also shown in the main body.

Table S5. ANN results for all test samples.

ANN	Sample ID											
Output	37	38	39	40	41	42	43	44	45	46	47	48
1	4.132	3.582	5.147	2.986	3.650	4.303	3.714	3.930	3.268	4.881	3.531	4.540
2	2.546	2.360	3.440	1.621	2.266	2.739	2.058	2.454	1.960	2.818	1.486	2.750
3	1.983	1.800	2.702	1.187	1.715	2.114	1.544	1.891	1.389	2.119	0.962	2.281
4	1.660	1.520	2.283	0.926	1.433	1.769	1.265	1.583	1.121	1.734	0.666	1.957
5	1.449	1.320	1.989	0.772	1.191	1.541	1.049	1.389	0.921	1.473	0.538	1.789
6	1.332	1.172	1.812	0.655	1.060	1.413	0.915	1.262	0.782	1.330	0.428	1.723
7	1.230	1.035	1.657	0.567	0.966	1.293	0.804	1.137	0.679	1.209	0.324	1.658
8	1.119	0.944	1.501	0.506	0.868	1.202	0.727	1.063	0.602	1.071	0.314	1.581
9	1.041	0.873	1.412	0.459	0.795	1.120	0.655	0.986	0.544	1.004	0.266	1.503
10	0.970	0.806	1.312	0.418	0.728	1.030	0.601	0.906	0.499	0.904	0.238	1.428
11	0.924	0.755	1.249	0.378	0.682	0.974	0.557	0.853	0.459	0.853	0.204	1.388
12	0.865	0.702	1.169	0.345	0.637	0.919	0.515	0.804	0.422	0.792	0.183	1.348
13	0.820	0.661	1.114	0.323	0.600	0.874	0.475	0.760	0.396	0.748	0.162	1.306
14	0.786	0.631	1.073	0.303	0.572	0.844	0.448	0.732	0.371	0.718	0.146	1.279

15	0.747	0.602	1.018	0.287	0.543	0.811	0.427	0.706	0.347	0.673	0.141	1.253
16	0.717	0.576	0.973	0.273	0.516	0.784	0.405	0.684	0.328	0.634	0.140	1.236
17	0.691	0.553	0.941	0.261	0.493	0.755	0.385	0.658	0.314	0.606	0.134	1.214
18	0.659	0.529	0.905	0.250	0.475	0.725	0.369	0.630	0.303	0.574	0.130	1.186
19	0.636	0.510	0.879	0.240	0.456	0.701	0.354	0.608	0.291	0.550	0.126	1.169
20	0.618	0.491	0.857	0.231	0.441	0.677	0.345	0.587	0.279	0.533	0.122	1.158
21	0.602	0.477	0.837	0.224	0.430	0.662	0.336	0.573	0.269	0.517	0.118	1.152
22	0.591	0.466	0.822	0.217	0.420	0.653	0.326	0.565	0.259	0.506	0.113	1.153
23	0.582	0.459	0.806	0.213	0.411	0.650	0.316	0.564	0.250	0.494	0.110	1.158
24	0.567	0.449	0.791	0.209	0.402	0.636	0.307	0.552	0.244	0.481	0.105	1.151
25	0.554	0.439	0.777	0.205	0.391	0.621	0.299	0.539	0.240	0.464	0.105	1.143
26	0.542	0.430	0.765	0.201	0.382	0.606	0.294	0.526	0.236	0.450	0.105	1.136
27	0.530	0.421	0.754	0.198	0.375	0.591	0.290	0.513	0.233	0.440	0.103	1.130
28	0.525	0.419	0.747	0.194	0.368	0.590	0.283	0.514	0.228	0.430	0.104	1.137
29	0.522	0.415	0.744	0.191	0.361	0.585	0.277	0.510	0.224	0.426	0.102	1.144
30	0.518	0.409	0.742	0.188	0.357	0.577	0.274	0.503	0.221	0.423	0.100	1.149
31	0.518	0.407	0.739	0.186	0.354	0.575	0.272	0.504	0.217	0.421	0.100	1.161
32	0.519	0.405	0.740	0.184	0.352	0.572	0.273	0.502	0.214	0.423	0.099	1.169
33	0.517	0.401	0.744	0.183	0.352	0.561	0.276	0.493	0.213	0.426	0.097	1.167
34	0.515	0.399	0.748	0.182	0.351	0.556	0.279	0.489	0.213	0.425	0.098	1.165
35	0.519	0.400	0.756	0.181	0.350	0.557	0.279	0.490	0.211	0.430	0.097	1.167
36	0.524	0.402	0.768	0.182	0.351	0.561	0.278	0.494	0.210	0.438	0.093	1.169
37	0.528	0.406	0.783	0.182	0.351	0.569	0.277	0.501	0.210	0.443	0.093	1.164
38	0.530	0.409	0.799	0.183	0.352	0.573	0.279	0.503	0.211	0.449	0.095	1.151
39	0.534	0.414	0.813	0.183	0.354	0.586	0.280	0.515	0.212	0.451	0.100	1.144
40	0.537	0.420	0.825	0.185	0.358	0.601	0.283	0.528	0.214	0.452	0.107	1.138
41	0.547	0.432	0.843	0.188	0.366	0.630	0.284	0.553	0.216	0.457	0.111	1.139
42	0.555	0.441	0.866	0.191	0.374	0.650	0.290	0.568	0.220	0.465	0.116	1.129
43	0.563	0.450	0.888	0.194	0.380	0.664	0.298	0.579	0.224	0.474	0.122	1.120
44	0.576	0.460	0.915	0.197	0.386	0.679	0.306	0.591	0.228	0.487	0.128	1.116
45	0.584	0.472	0.940	0.203	0.394	0.693	0.316	0.601	0.234	0.494	0.135	1.101
46	0.593	0.483	0.965	0.210	0.401	0.705	0.327	0.611	0.241	0.501	0.143	1.086
47	0.599	0.495	0.998	0.216	0.409	0.714	0.340	0.615	0.250	0.510	0.152	1.062
48	0.611	0.509	1.025	0.225	0.419	0.731	0.352	0.629	0.258	0.522	0.161	1.057
49	0.624	0.523	1.065	0.234	0.432	0.740	0.370	0.633	0.270	0.543	0.167	1.039
50	0.641	0.542	1.105	0.245	0.451	0.757	0.392	0.644	0.286	0.563	0.173	1.026
51	0.662	0.561	1.148	0.257	0.471	0.769	0.418	0.651	0.303	0.591	0.182	1.021
52	0.691	0.586	1.196	0.270	0.494	0.787	0.450	0.667	0.319	0.629	0.192	1.029
53	0.719	0.611	1.247	0.285	0.514	0.805	0.478	0.682	0.333	0.668	0.202	1.037
54	0.739	0.636	1.289	0.304	0.539	0.818	0.514	0.694	0.354	0.697	0.216	1.034
55	0.768	0.665	1.345	0.328	0.571	0.833	0.553	0.706	0.379	0.738	0.224	1.032
56	0.802	0.703	1.394	0.356	0.602	0.857	0.591	0.732	0.406	0.777	0.241	1.050
57	0.845	0.751	1.453	0.385	0.637	0.899	0.634	0.775	0.437	0.826	0.268	1.083
58	0.902	0.812	1.518	0.420	0.680	0.949	0.687	0.830	0.475	0.886	0.303	1.135
59	0.961	0.873	1.602	0.462	0.741	1.015	0.753	0.893	0.517	0.967	0.338	1.195
60	1.044	0.949	1.710	0.509	0.800	1.079	0.830	0.959	0.567	1.070	0.389	1.274
61	1.147	1.055	1.829	0.563	0.885	1.183	0.921	1.065	0.640	1.181	0.452	1.379
62	1.253	1.179	1.964	0.643	0.983	1.303	1.025	1.190	0.727	1.312	0.529	1.482
63	1.436	1.327	2.145	0.765	1.113	1.478	1.165	1.362	0.833	1.489	0.659	1.704
64	1.644	1.532	2.388	0.909	1.300	1.707	1.352	1.585	0.989	1.722	0.804	1.925
65	1.977	1.839	2.743	1.152	1.521	2.018	1.640	1.915	1.211	2.072	1.135	2.319
66	2.475	2.409	3.343	1.551	1.963	2.532	2.156	2.463	1.686	2.670	1.654	2.747
67	4.132	3.582	5.147	2.986	3.650	4.303	3.714	3.930	3.268	4.881	3.531	4.540

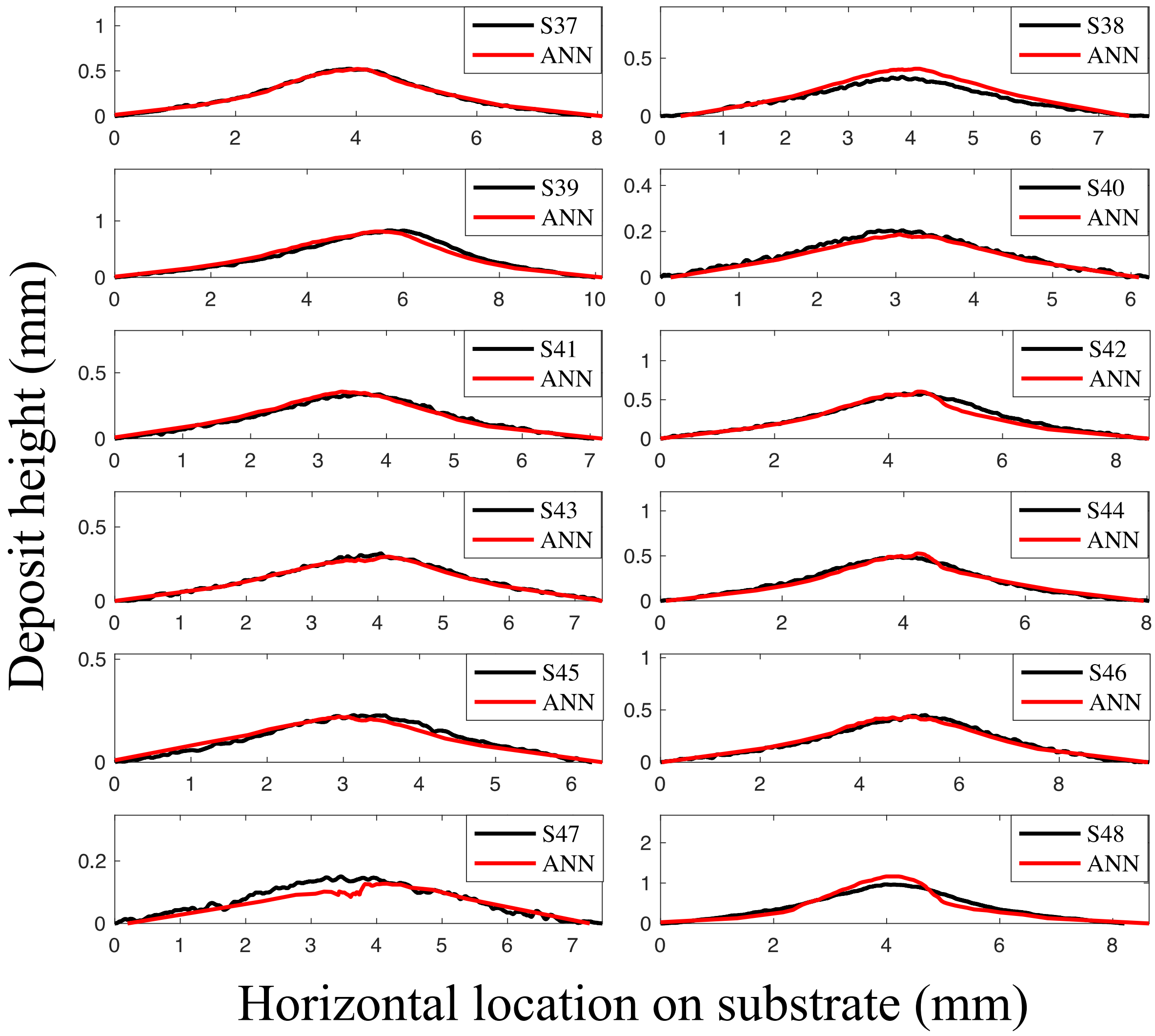

Figure S4. The predicted track profiles (solid red line) using the developed ANN model. The measured profiles are also presented for comparison (solid black line). Sample 37 and Sample 39 are also shown in the main body.

Gaussian model details

In Figure 4 presented in the main body, we adopted a mathematical Gaussian modelling approach proposed by Chen *et al.* in cold spray [1]. In this method, three parameters were changed: (1) scaling constant to adjust the height of the model, (2) mean location along the x-axis (or on substrate) and (3) standard deviation to determine the shape of the model. The details of each parameter value for Sample 37 and 39 are listed in Table S6. Note that the standard deviations are the values of those prior spray angle corrections as explained in section 2.1 in [1]. A number of parameter sets were tested to find the model that fitted best with the experimentally measured profiles.

Table S6. Gaussian model parameters.

Sample ID	Scaling Constant	Mean (mm)	Standard Deviation
37	1.04	3.878	1.3
39	1.50	4.996	1.2

References

1. Chen, C.; Xie, Y.; Verdy, C.; Liao, H.; Deng, S. Modelling of coating thickness distribution and its application in offline programming software. *Surf. Coat. Tech.* **2017**, *318*, 315–325.

© 2019 by the authors. Submitted for possible open access publication under the terms and conditions of the Creative Commons Attribution (CC BY) license (http://creativecommons.org/licenses/by/4.0/).
